# Repurposing non-antifungal drugs auranofin and pentamidine in combination as fungistatic antifungal agents against *C. albicans*

**DOI:** 10.3389/fcimb.2022.1065962

**Published:** 2022-12-15

**Authors:** Jiaying Lin, Xueyi Xiao, Yijing Liang, Huimin Zhao, Yingxiao Yu, Peiyan Yuan, Sha Lu, Xin Ding

**Affiliations:** ^1^ School of Pharmaceutical Sciences (Shenzhen), Shenzhen Campus of Sun Yat-sen University, Shenzhen, Guangdong, China; ^2^ Department of Dermatology, Sun Yat-sen Memorial Hospital, Sun Yat-sen University, Guangzhou, China

**Keywords:** drug repurposing, auranofin, pentamidine, combination treatment, antifungals

## Abstract

Fungal infection is a serious global health issue, causing approximately 1.5 million mortalities annually. However, clinically available anti-fungal drugs are limited, especially for multidrug-resistant fungal infections. Therefore, new antifungal drugs are urgently needed to address this clinical challenge. In this study, we proposed two non-antifungal drugs, auranofin and pentamidine, in combination to fight against multidrug-resistant *C. albicans*. The insufficient antifungal activity of anti-rheumatic drug auranofin is partially due to fungal membrane barrier preventing the drug uptake, and anti-protozoal drug pentamidine was used here to improve the permeability of membrane. The auranofin/pentamidine combination displayed synergistic inhibitory effect against both drug-susceptible and drug-resistant *C. albicans*, as well as biofilm, and significantly reduced the minimum inhibitory concentration of each drug. At non-antifungal concentration, pentamidine can disrupt the membrane integrity and increase membrane permeability, leading to enhanced cellular uptake of auranofin in *C. albicans*. This repurposing strategy using the combination of non-antifungal drugs with complementary antifungal mechanism may provide a novel approach for discovery of antifungal drugs to fight against multidrug-resistant fungal infections.

## Introduction

Fungal infections affect over 25 percent of the population, and kill approximately 1.5 million people annually (Brown et al., 2012; Rodrigues and Albuquerque, 2018). More importantly, fungal pathogens have shown resistance to a variety of antifungal drugs through mechanisms such as promoted efflux, thickened biofilm and target mutation (Vandeputte et al., 2012). For example, 93% of *Candida* isolates showed resistance to fluconazole and 35% *Candida* isolates were resistant to amphotericin B (Lockhart et al., 2016). There is an urgent need to develop new antifungal drugs for treatment of resistant fungal infections. Drug repurposing strategy is of great value owing to the available physicochemical attributes and pharmacokinetic properties of drugs on the market, and this approach could greatly reduce the cost and time of new drug development. The repurposed drugs with mechanism of action differing from current anti-fungal drugs would be promising to address the problem of multidrug resistance. At present, the anti-fungal activity and action of mechanism of various drugs such as anthelmintic, anti-cancer and anti-inflammatory drugs have been studied (Miró et al., 2019). Several drugs have been also showed synergistic antifungal activity when they are co-applied with antifungal drugs. However, there is no repurposed drugs, to the best of our knowledge, that have been clinically used as antifungals, mainly due to the insufficient efficacy in controlling fungal pathogens and safety issue.

**Figure 1:**
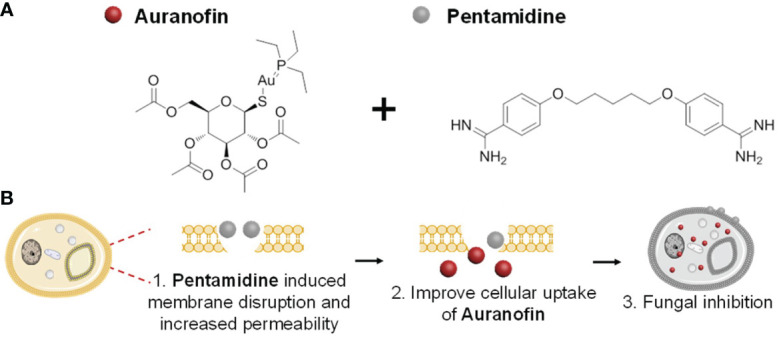
**(A)** The chemical structures of combined drugs auranofin (left) and pentamidine (right), and **(B)** proposed synergistic antifungal mechanism of the drug combination.

Auranofin (**Figure 1**) is a drug approved by FDA for the treatment of rheumatoid arthritis (Chaffman et al., 1984). Recently, auranofin has been determined to possess antibacterial and antifungal activity both used alone and in combination with antibiotics. In particular, auranofin is capable of inhibiting the growth of *Candida*, *Cryptococcus* and other few fungi *in vitro* (Wiederhold et al., 2017), and the antifungal effect could be associated with preventing Mia40 from binding to its substrate Cmc1 and subsequently depressing the oxidation of cysteine-rich proteins in fungal mitochondria (Thangamani et al., 2017). Auranofin can also irreversibly bind with thiol and selenol groups of thioredoxin reductase in fungal mitochondria, which diminishes the protective ability against reactive oxygen species produced in respiratory process, leading to the damage of macromolecules caused by oxidative stress in fungal cells (May et al., 2018). However, the antifungal activity of auranofin is unsatisfactory for further clinical application. Wiederhold et al. have tested the minimal inhibitory concentration (MIC) of auranofin against various clinically-isolated *C. albicans*, and found that most MIC values were ≥16.0 μg/mL which was much higher than that of clinical-used antifungal drug fluconazole (MIC: 0.25 – 8.0 μg/mL) (Wiederhold et al., 2017). Therefore, the anti-fungal activity of auranofin need to be further improved before it can be successfully repurposed for treatment of fungal infections.

Pentamidine (**Figure 1**) is an antiprotozoal drug commonly used in the treatment of trypanosomiasis and leishmaniasis (Brendle et al., 2002). The inhibitory activity of pentamidine against various fungi such as *Pneumocystis carinii* (Sands et al., 1985), *Candida albicans* (Miletti and Leibowitz, 2000), *Sporothrix schenckii (* Brilhante et al., 2018) and pathogenic *Fusarium (* Venturini et al., 2016) *in vitro* has been reported. The antifungal activity of pentamidine could be associated with inhibition of DNA, RNA, phospholipid and protein synthesis (Rossato et al., 2021), suppression of splicing of rRNA intron in yeast mitochondria (Zhang et al., 2000), and interference with tRNA structure during translation (Sun and Zhang, 2008). Although pentamidine has been clinically used to treat pneumocystis pneumonia in HIV-infected patients over 30 years (Sands et al., 1985), this drug has not been used for treatment of other fungal infections, probably due the insufficient anti-fungal activity. Recently, it was reported that pentamidine exhibits synergistic antibacterial effect on a variety of Gram-negative drug-resistant bacteria in combination with antibiotics, as it can disrupt bacterial membrane and result in increased membrane permeation to antibiotics (Herrera-Espejo et al., 2020; Stokes et al., 2017).

Inspired by the aforementioned work which use pentamidine to potentiate the membrane permeability of traditional antibiotic, we proposed of combining auranofin with pentamidine, aiming to improve their anti-fungal activity. Pentamidine was selected in this study to enhance the uptake of auranofin in fungal cells by disrupting cell membrane, leading to stronger antifungal activity of auranofin. Meanwhile, the multimode anti-fungal mechanisms of auranofin and pentamidine may reduce the chance of resistance development in fungal cells when compared with drug mono-treatment. Herein, the anti-fungal activity of the drug combination against drug-susceptible and drug-resistant *C. albicans* was examined, and the mechanism of action was then explored.

## Materials and methods

### Organisms, cultures and antifungals

A drug sensitive strain of *C. albicans* (64548) and a fluconazole-resistant strain (64550) were obtained from American Type Culture Collection (ATCC), and a clinical isolate of multi-drug resistant *C. albicans* (*C. albicans*-1, *C. albicans*-2 and *C. albicans*-3) was supplied by Sun Yat-Sen Memorial Hospital (Guangzhou, China). All the strains had been previously identified as *C. albicans* on the basis of morphology and molecular identification. Sterilized Yeast Extract-Malt Extract Broth (YMB, BD Difco, USA) and Luria-Bertani (LB) broth (BD Difco, USA) were used as fungal liquid culture and agar solid culture, respectively. Auranofin was purchased from Abcam (Shanghai) Trade Co., Ltd. (Shanghai, China) and pentamidine was purchased from Melone Pharmaceutical (Dalian) Co., Ltd. (Dalian, China). Auranofin and pentamidine were dissolved in dimethyl sulfoxide and sterile distilled water to prepare the stock solution, respectively.

### Chequerboard broth microdilution assays

Chequerboard assays were performed according to CLSI M27-A3 guideline with serial-diluted auranofin and pentamidine (Wayne and Clinical and Laboratory Standards Institute, 2008). Overnight culture of fungi (~10^4^ CFU/mL) was added to all wells in chequerboards. The chequerboards were incubated at 25°C for 48 h and the optical density values at 600 nm (OD_600_) of each well were detected by microplate reader at 0 h and 48 h. Each chequerboard assay was performed in triplicate.

The synergistic effect of auranofin and pentamidine was quantitatively evaluated by the fractional inhibitory concentration index (FICI), which was calculated according to the formula $FICI=\frac{MIC_{A-combined}}{MIC_{A-alone}}+\frac{MIC_{B-combined}}{MIC_{B-alone}}$ . FICI ≤ 0.5 represents synergism of two drugs. No interaction occurred at 0.5< FICI< 4.0 and antagonism occurred at FICI ≤ 4.0 (Odds, 2003).

### Growth inhibition and time-killing kinetics tests

The OD_600_ values of each well in the chequerboards aforementioned were measured at specific time points during incubation. Growth curves were plotted with representative OD_600_ values. Each test was performed in triplicate.

Samples (10 μL) collected from wells of the aforementioned chequerboards were plated on LB broth agar after dilution in phosphate-buffered saline (PBS). The colonies on the plates were counted to calculate colony forming units per milliliter (CFU/mL) of inoculum after being cultured at 37°C overnight, which were used to plot time-killing curves. Each test was performed in triplicate.

### Anti-biofilm activity characterized by confocal laser scanning microscopy and crystal violet staining

The *C. albicans* (ATCC-64550) (~10^4^ CFU/mL) were treated with pentamidine (7.8 μg/mL), auranofin (2.0 μg/mL) or their combination in the confocal dishes without shaking at 37°C for 48 h. *C. albicans* without treatment was used as control group. The fungi were then stained with the LIVE/DEAD Baclight viability kit (Thermo Fisher Scientific, America) using manufacture’s protocol. The 3D images were captured using a two-photon confocal laser scanning microscope (Carl Zeiss, Germany). To quantify biomass level, crystal violet assay was performed. *C. albicans* were treated with same condition as confocal study, and then the crystal violet (100 μL, 1%, w/v) was incubated with biofilm samples at room temperature for 40 min. Subsequently, the crystal violet solution was removed and followed by adding ethanol (200 μL) in each well. After shaking for 30 min, the absorbance at 595 nm was measured using a microplate reader. Each test was performed in triplicate.

### Membrane permeability characterized by CLSM and fluorospectrophotometer

The *C. albicans-3* growing at exponentially phase (~10^7^ CFU/mL) were treated with pentamidine (15.6 μg/mL or 31.3 μg/mL). After 2 h of incubation at 25°C, the fungi were harvested through centrifugation at 4°C, 5000 rpm for 10 mins and washed with 0.9% NaCl saline twice. The fungi in different treatment groups were subsequently stained using the LIVE/DEAD Baclight viability kit (Thermo Fisher Scientific, America). Fluorescence was observed using a two-photon confocal laser scanning microscope (Carl Zeiss, Germany). The fluorescence intensity of propidium iodide was detected quantitatively using fluorospectrophotometer (Perkin-Elmer, America). Quantification assay was performed in triplicate.

### Scanning electron microscopy imaging

The *C. albicans* growing at exponentially phase (~10^7^ CFU/mL) were treated with pentamidine (31.3 μg/mL) for 2 h at 25°C. After that, the fungi were harvested through centrifugation at 4°C, 3500 rpm for 6 mins and washed with PBS twice. Paraformaldehyde dissolved in PBS solution (2.5%, 1 mL) was used for fixation of the treated fungi cells, and the samples were kept at 4°C overnight. The fixed samples were washed with PBS and distilled water twice, followed by 15 min dehydration each in ethanol solutions of 50%, 75%, 90% and twice in 100%. Afterwards, samples were freeze-dried, gold-sprayed, conducting resin-adhered and observed under the scanning electron microscopy (Carl Zeiss, Germany).

### Intracellular gold element analysis

The *C. albicans* growing at exponentially phase (~10^7^ CFU/mL) were treated with auranofin (31.3 μg/mL or 62.5 μg/mL) together with pentamidine 31.3 μg/mL. Upon 1 h of incubation at 25°C, fungi were harvested through centrifugation at 4°C, 5000 rpm for 10 mins and washed with PBS twice. Subsequently, fungal cells were crushed by ultrasonic cell disruptor for 10 mins, and -20°C acetone was then added to precipitate protein. After precipitation, samples were centrifuged at 4°C, 13500 rpm for 1 min and the supernatant was discarded. The gold element content of samples was detected by inductively coupled plasma-mass spectrometry (ICP-MS, Thermo Fisher Scientific, USA). Each analysis was performed in triplicate.

### Hemolysis level

Fresh mice blood was diluted 25-fold with sterile PBS, and then the diluted blood (250 μl) was mixed with PBS containing auranofin and pentamidine at various concentrations (i.e., auranofin 62.5 μg/ml + pentamidine ranging from 0 to 125μg/ml pentamidine; pentamidine 62.5 μg/ml + auranofin ranging from 0 to 125μg/ml pentamidine). PBS only and Triton (0.1%, v/v) was mixed with the diluted blood as negative and positive control, respectively. The samples were incubated at 37°C for 1h, and followed by centrifugation at 2200 rpm for 5 min. The hemolysis pictures were taken and each sample from the supernatant (100 μl) was placed in each well of a 96-well plate. The absorbance at 490 nm measured with a microplate reader was obtained to calculate hemolysis ratio (%) using the following equations:


$$((OD_{s}-OD_{nc})/(OD_{pc}-OD_{nc}))\times 100\%$$

OD_s_: OD_490_ values for samples, OD_nc_: OD_490_ values for negative controls, OD_pc_: OD_490_ values for positive controls.

## Results

### Synergistic anti-fungal effect of auranofin and pentamidine

Chequerboard assays were performed to evaluate synergistic effect between auranofin and pentamidine against drug-sensitive standard strain, fluconazole-resistant standard strain and clinical multi-drug resistant strain of *C. albicans.* The results of chequerboard assays showed that auranofin and pentamidine possessed synergistic anti-fungal effect against all the *C. albicans* tested (**Table 1**). For the drug-sensitive *C. albicans*, the MIC of auranofin and pentamidine alone were 15.6 μg/mL and 125 μg/mL, respectively, implying the weak anti-fungal activity for monotherapy. However, the growth was totally inhibited in the presence of auranofin at 2.0 μg/mL combined with pentamidine at 7.8 μg/mL, and the fractional inhibitory concentration was determined to be 0.19, indicating strong synergistic interaction of auranofin and pentamidine against this drug-sensitive strain of *C. albicans* (**Table 1**). The similar synergistic effect was observed for fluconazole-resistant strain of *C. albicans* (ATCC 64550). In addition, three more clinically isolated *C. albicans* strains obtained from Sun Yat-sen Memorial Hospital have been further tested to verify antifungal activity of the combination. The FICI obtained for all the three strains are lower than 0.50 (**Table 1**), indicating the synergistic effect of auranofin and pentamidine against clinically isolated strains. Surprisingly, the clinically isolated *C. albicans-3* also exhibited resistance to drug auranofin, as the MIC of auranofin was 125 μg/mL which was significantly higher than drug susceptible strains of *C. albicans.* However, the MIC of auranofin after combining with pentamidine reduced to 31.3 μg/mL, which is similar to the MIC of auranofin against drug-susceptible strain. In short, the MICs of auranofin and pentamidine in combination against drug-susceptible and drug-resistant *C. albicans* were lower than those drugs used alone. The FICI of the combination in these *C. albicans* strains were all lower than 0.50, indicating synergism of auranofin and pentamidine.

**Table 1:** MIC of auranofin and pentamidine alone and in combination, and FICI values of the combination against *C. albicans*.

Organism	AuranofinMIC_combined_/ MIC_alone_ (μg/mL)	PentamidineMIC_combined_/MIC_alone_ (μg/mL)	FIC index	Interpretation
*C. albicans ATCC 64548*	2.0 / 15.6	7.8 / 125	0.19	synergy
C. albicans ATCC 64550*	2.0 / 7.8	7.8 / 31.3	0.50	synergy
*C. albicans-1^#^*	0.9 / 3.9	7.8 /62.5	0.36	synergy
*C. albicans-2^#^*	2.0 /15.6	15.6 /125	0.25	synergy
*C. albicans-3* ^#^	31.3 / 125	3.9 / >15.6	<0.50	synergy

*drug-resistant C. albicans. ^#^clinically isolated C. albicans strains. MIC, minimum inhibitory concentration; FICI, fractional inhibitory concentration index. $FICI=\frac{MIC_{A-combined}}{MIC_{A-alone}}+\frac{MIC_{B-combined}}{MIC_{B-alone}}$ and FICI ≤ 0.500 represents synergism of two drugs.

**Figure 2:**
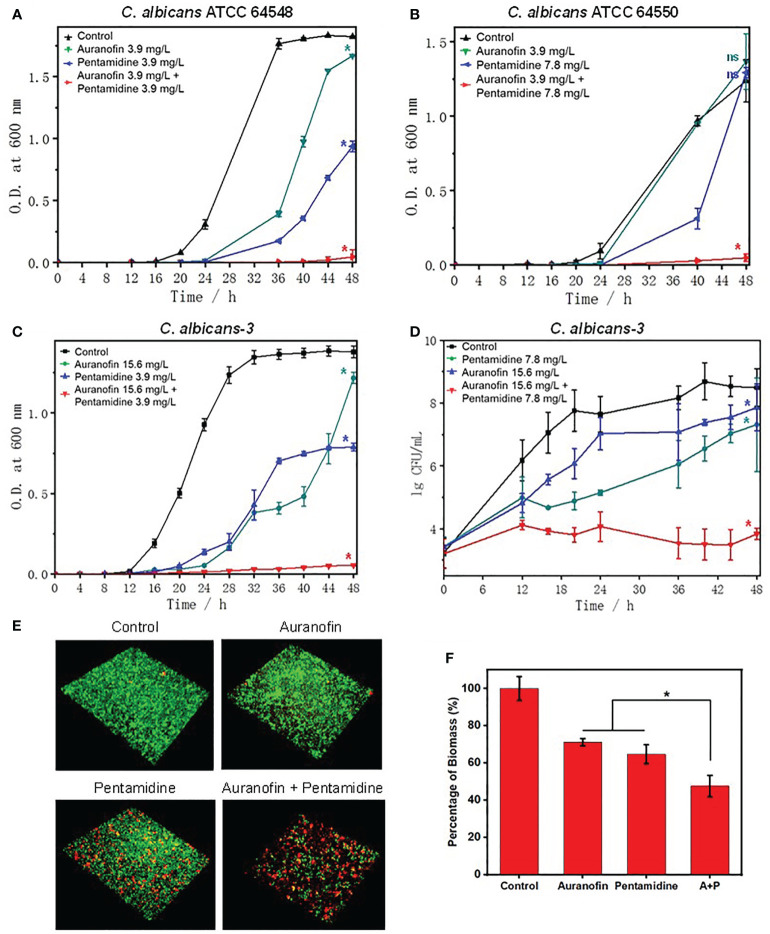
Anti-fungal and anti-biofilm activity of the combination of auranofin and pentamidine against *C albicans*. **(A)** Growth curve of *C. albicans* ATCC 64548, a drug-susceptible standard strain. **(B)** Growth curve of *C. albicans* ATCC 64550, a fluconazole-resistant standard strain. **(C)** Growth curve of *C. albicans*-3, a multidrug-resistant strain. **(D)** Time-killing curve of *C. albicans*-3. The initial concentration of all fungi was ~5×10^3^ CFU/ml. **(E)** Representative 3D confocal images of *C. albicans* biofilms obtained after different treatments (auranofin: 2.0 μg/mL; pentamidine: 7.8 μg/mL; combination of auranofin and pentamidine at the same concentration of mono-treatment) for two days. **(F)** Percentage of biomass of *C. albicans* biofilm characterized by crystal violet staining under the same treatment conditions as **(E)**. (n = 3, statistical analysis was conducted using Student’s t-test, *p< 0.05, ns represents no significant difference between treatment group and control group).

To further study the antifungal activity of the drug combination, grow inhibition kinetics and time-killing kinetics were investigated. As shown in **Figures 2A–C**, the combination of auranofin and pentamidine achieved virtually complete inhibition against all the tested susceptible and resistant strains of *C. albicans* over 48 h of incubation, and the concentration of each drug used in combination was lower than their MIC measured in mono-treatment. Apparently, the two drugs used alone at the identical concentration could not totally prevent the growth of *C. albicans*, only showing slight inhibitory effect and delayed growth curve. The time-killing kinetics against clinically isolated drug-resistant *C. albicans* revealed that growth inhibition was due to the fungistatic activity of the combination of auranofin and pentamidine (**Figure 2D**). The number of *C. albicans* incubated with drug combination almost kept constant, while growth of *C. albicans* was not significantly affected by the mono-treatment of pentamidine or auranofin. Although the drug combination did not kill *C. albicans*, it could be used as fungistatic drugs similar as azole antifungal drugs such as fluconazole and ketoconazole (Lewis and Graybill, 2008).

As the combination of auranofin and pentamidine can inhibit the growth of *C. albicans*, the anti-biofilm activity was further investigated. The confocal images of live (green)/dead (red) staining kit-stained *C. albicans* showed that the drug combination was capable of preventing the formation of mature biofilm, while dense biofilms were observed for the untreated or mono-drug treated *C. albicans* after culturing for 48 h (**Figure 2E**). Moreover, the biomass level of biofilm characterized by crystal violet staining also verified that the drug combination could inhibit biofilm formation (**Figure 2F**).

### Effect of pentamidine on membrane permeability of *C. albicans*

To explore the mechanism for the synergistic antifungal activity of auranofin/pentamidine combination, the membrane permeability of *C. albicans* upon pentamidine treatment was studied. As pentamidine was identified as an effective perturbant of the Gram-negative outer membrane through its interaction with negatively charged lipopolysaccharide, and increase membrane permeability to antibiotics (Ando et al., 2012; Stokes et al., 2017). Considering that the fungal surface is covered with a linear-branched network structure composed of negatively charged phosphate groups (Werner et al., 2007), here we suspected that pentamidine produced a similar membrane disruption effect on *C. albicans* and altered the membrane permeability.

**Figure 3:**
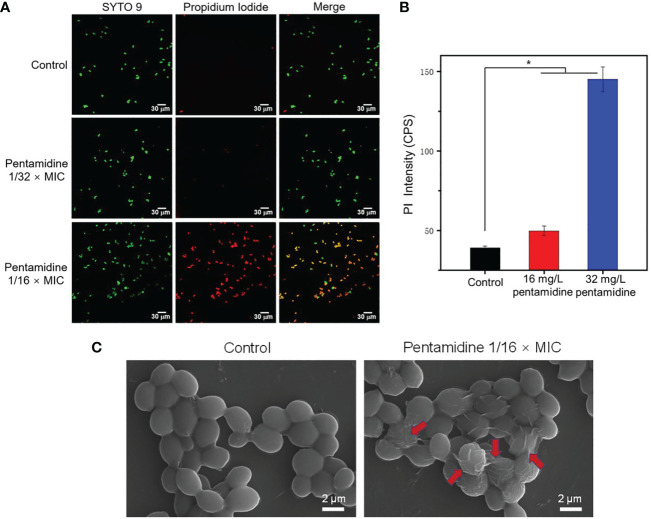
Pentamidine increased the cell permeability and disrupted the surface structure of *C. albicans*-3. **(A)** The confocal laser scanning microscopic (CLSM) images *C. albicans* of *C. albicans*-3 under different treatments stained with SYTO 9 (green) and PI (red) (scale bar: 30 μm). **(B)** The fluorescence intensity of PI in *C. albicans*-3 detected by fluorospectrophotometer. The data were representative of three biological replicates. **(C)** The scanning electron microscopic (SEM) images of *C. albicans*-3 treated with pentamidine at 1/16 × MIC. Red arrows point to the disrupted cell membrane. (n = 3, statistical analysis was conducted using Student’s t-test, *p< 0.05).

In order to visualize the fungal cell permeability, a LIVE/DEAD staining kit was used to stain fungal cells treated with pentamidine, which consists of a penetrable dye named SYTO 9 and a dye propidium iodide (PI) only penetrate the disrupted membrane. The fluorescence of stained cells was observed using a confocal laser scanning microscope (**Figure 3A**) and the fluorescence intensity of PI was quantified (**Figure 3B**). The fluorescent images showed that *C. albicans*-3 cells treated with pentamidine at 1/16 × MIC were greatly permeable to PI stain, which is in agreement with the significantly high fluorescence intensity of PI in these cells when compared with untreated cells and cells treated with pentamidine at lower concentration (1/32 × MIC). These data suggested that pentamidine at non-antifungal concentration can improve membrane permeability of clinically isolated multidrug-resistant *C. albicans*. In addition, surface morphology changes upon pentamidine treatment of *C. albicans* were characterized using scanning electron microscope (SEM). The results showed that the fungal surface became rough and winkled after incubation with pentamidine (1/16 × MIC) for 2 h, while untreated cells revealed a smooth cell surface (**Figure 3C**). The observation of surface morphology indicated that pentamidine produced a cell surface disruption effect and enabled *C. albicans* cells to lose membrane integrity, resulting in increasing membrane permeability.

### Effect of pentamidine on intracellular auranofin content

**Figure 4:**
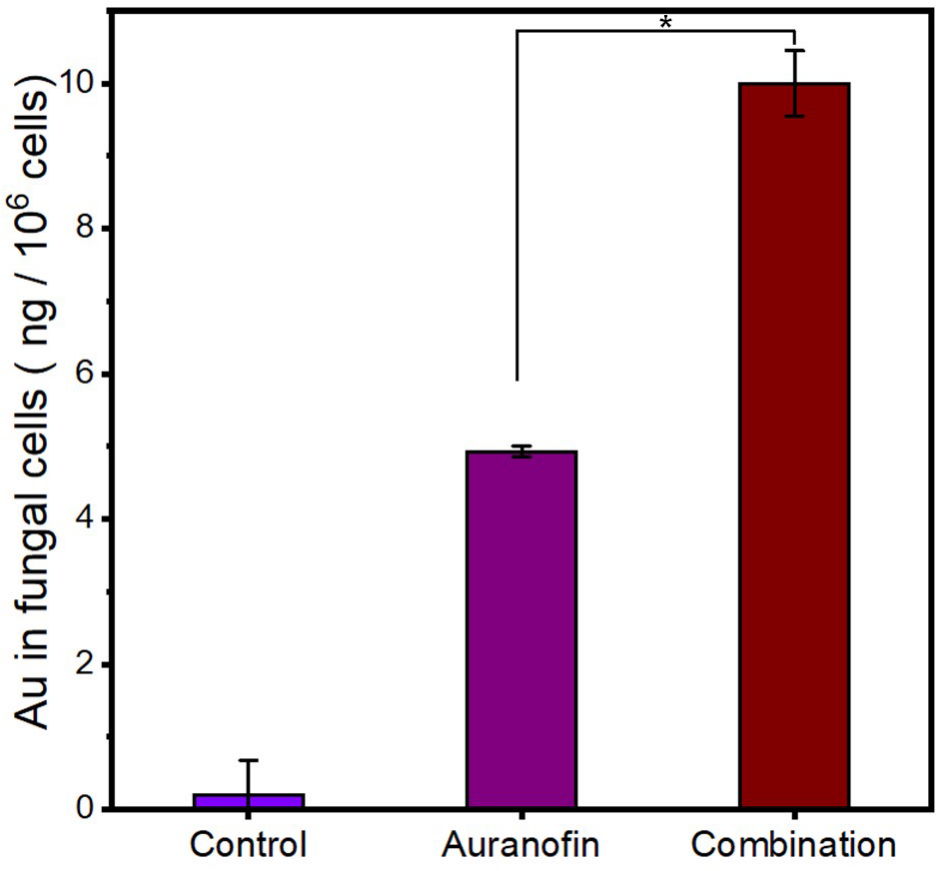
The increased intracellular content of auranofin in *C. albicans*-3 cells in the presence of pentamidine at 1/16 × MIC. Control: cells with no treatment. Auranofin: cells treated with auranofin for 1 h. Combination: cells treated with auranofin (62.5 μg/mL) in the presence of pentamidine for 1 h. (n = 3, statistical analysis was conducted using Student’s t-test, *p< 0.05).

Considering the surface disruption and permeability increase of *C. albicans*’ membrane upon pentamidine treatment, we hypothesize that auranofin could be easier to be internalized by fungal cells when combined with pentamidine, leading to synergistic effect. The intracellular content of auranofin was tested by quantifying Au element in fungal cells treated with gold-containing auranofin in combination with pentamidine using inductively coupled plasma-mass spectrometry (ICP-MS). As shown in **Figure 4**, the amount of intracellular auranofin under combination treatment was almost twice as much as that under auranofin mono-drug treatment. This result demonstrated that pentamidine improved the membrane permeability of *C. albicans* and subsequently enhanced the uptake of auranofin in fungal cells.

It’s reported that antifungal targets of auranofin are located in the fungal cell, such as Mia40 which is associated with mitochondrial respiration (Thangamani et al., 2017). Therefore, increased intracellular auranofin content could correlate with promoted antifungal activity of auranofin, explaining why antifungal efficacy of auranofin in combination with pentamidine was better than that of auranofin used alone. In addition to the disruption effect of pentamidine on cell membrane, the antifungal mechanisms of pentamidine are associated with inhibition of mitochondrial rRNA splicing, rRNA translation and other intracellular actions (Zhang et al., 2000; Sun and Zhang, 2008). Thus, auranofin and pentamidine produced their antifungal effects by acting on different targets, which may be also related to synergistic antifungal effect of the combination. Furthermore, the multiple drug targets of the combination may also reduce the possibility of drug resistance when compared to drug with only one target.

### Hemocompatibility of the drug combination

**Figure 5:**
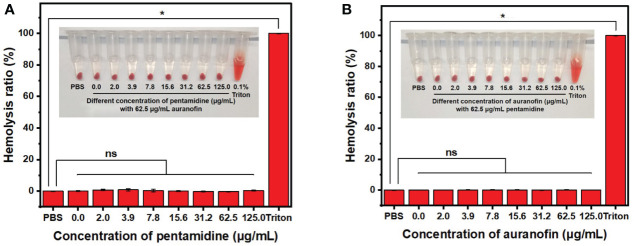
Hemolysis assay of drug combination auranofin/pentamidine using mouse blood. **(A)** The hemolysis picture (inset) and hemolysis ratio of the combination of auranofin (62.5 μg/mL) and pentamidine (concentration ranging from 0 to 125.0 μg/mL). **(B)** The hemolysis picture (inset) and hemolysis ratio of the combination of pentamidine (62.5 μg/mL) and auranofin (concentration ranging from 0 to 125.0 μg/mL). (n = 3, statistical analysis was conducted using Student’s t-test. *p< 0.05, ns represents no significant difference between the treatment group with control group).

Hemolysis tests were conducted to evaluate the hemocompatibility of the combination of auranofin and pentamidine (**Figure 5**). In the presence of auranofin at 62.5 μg/mL which is higher than the MIC of auranofin in combination against all the tested *C. albicans* (2.0 – 31.3 μg/mL, **Table 1**), pentamidine even up to 125 μg/mL did not cause hemolysis. The similar hemocompatibility was also observed for the combination with pentamidine concentration fixed at 62.5 μg/mL and auranofin concentration ranged from 2.0 μg/mL to 125 μg/mL. The hemolysis assay showed that the combination of auranofin and pentamidine at effective fungistatic concentrations is hemocompatible, and the combination is also not hemolytic even at higher concentration up to 125 μg/mL (pentamidine) and 62.5 (auranofin) or the other way around. Although further *in vitro* and *in vivo* biosafety needed to be evaluated, the good hemocompatibility indicates that this drug combination may be used for treatment of systematic fungal infections.

## Conclusions

In this study, the non-antifungal drugs auranofin and pentamidine with complementary antifungal mechanisms were repurposed by combination for inhibition of *C. albicans*. The chequerboard assay proved the synergistic antifungal effect of auranofin and pentamidine (FICI< 0.50), and the combination significantly lowered the MIC of both drugs against drug-susceptible, fluconazole-resistant and clinically isolated multi-drug resistant *C. albicans*. Grow inhibition kinetics and time-killing kinetics revealed that the combination of auranofin and pentamidine acted as fungistatic drugs that could totally inhibit the growth of *C. albicans*. The enhanced antifungal activity was associated with the membrane disruption and increased permeability caused by pentamidine, resulting in higher auranofin uptake in fungal cells. This repurposing strategy using the combination of non-antifungal drugs instead of antibiotics may provide a novel approach for discovery of antifungal drugs to fight against multidrug-resistant fungal infections.

## Data availability statement

The original contributions presented in the study are included in the article/supplementary material. Further inquiries can be directed to the corresponding authors.

## Author contributions

JL, XX and YL: Conducting a research and investigation process. HZ and YY: Maintain research data and formal analysis. PY, SL and XD: Presentation of the published work, specifically writing the draft, management and coordination responsibility for the research activity planning and execution. All authors approved the final manuscript. All authors contributed to the article and approved the submitted version.

## References

[B1] Ando M. Kamei R. Komagoe K. Inoue T. Yamada K. Katsu T. (2012). *In situ* potentiometric method to evaluate bacterial outer membrane-permeabilizing ability of drugs: Example using antiprotozoal diamidines. J. Microbiol. Meth 91, 497–500. doi: 10.1016/j.mimet.2012.09.033 23046554

[B2] Brendle J. J. Outlaw A. Kumar A. Boykin D. W. Patrick D. A. Tidwell R. R.. (2002). Antileishmanial activities of several classes of aromatic dications. Antimicrob. Agents Chemother. 46, 797–807. doi: 10.1128/AAC.46.3.797-807.2002 11850264 PMC127500

[B3] Brilhante R. S. N. Pereira V. S. Oliveira J. S. Lopes R. G. P. Rodrigues A. M. Camargo Z. P.. (2018). Pentamidine inhibits the growth of sporothrix schenckii complex and exhibits synergism with antifungal agents. Future Microbiol. 13, 1129–1140. doi: 10.2217/fmb-2018-0070 30113216

[B4] Brown G. D. Denning D. W. Gow N. A. R. Levitz S. M. Netea M. G. White T. C. (2012). Hidden killers: Human fungal infections. Sci. Transl. Med. 4, 165rv113. doi: 10.1126/scitranslmed.3004404 23253612

[B5] Chaffman M. Brogden R. N. Heel R. C. Speight T. M. Avery G. S. (1984). Auranofin. A preliminary review of its pharmacological properties and therapeutic use in rheumatoid arthritis. Drugs 27, 378–424. doi: 10.2165/00003495-198427050-00002 6426923

[B6] Herrera-Espejo S. Cebrero-Cangueiro T. Labrador-Herrera G. Pachón J. Pachón-Ibáñez M. E. Álvarez-Marín R. (2000). *In vitro* activity of pentamidine alone and in combination with antibiotics against multidrug-resistant clinical pseudomonas aeruginosa strains. Antibiotics 9, 885. doi: 10.3390/antibiotics9120885 PMC7764095 33317111

[B7] Lewis J. S. Graybill J. R. (2008). Fungicidal versus fungistatic: What's in a word? expert opin. Pharmacother 9, 927–935. doi: 10.1517/14656566.9.6.927 18377336

[B8] Lockhart S. R. Etienne K. A. Vallabhaneni S. Farooqi J. Chowdhary A. Govender N. P.. (2016). Simultaneous emergence of multidrug-resistant candida auris on 3 continents confirmed by whole-genome sequencing and epidemiological analyses. Clin. Infect. Dis. 64, 134–140. doi: 10.1093/cid/ciw691 27988485 PMC5215215

[B9] May H. C. Yu J. J. Guentzel M. N. Chambers J. P. Cap A. P. Arulanandam B. P. (2018). Repurposing auranofin, ebselen, and PX-12 as antimicrobial agents targeting the thioredoxin system. Front. Microbiol. 9, 336. doi: 10.3389/fmicb.2018.00336 29556223 PMC5844926

[B10] Miletti K. E. Leibowitz M. J. (2000). Pentamidine inhibition of group I intron splicing in candida albicans correlates with growth inhibition, antimicrob. Agents Chemother. 44, 958–966. doi: 10.1128/AAC.44.4.958-966.2000 PMC89798 10722497

[B11] Miró A. Ayerbe-Algaba R. Smani Y. (2019). Drug repurposing for the treatment of bacterial and fungal infections. Front. Microbiol. 10, 41. doi: 10.3389/fmicb.2019.00041 30745898 PMC6360151

[B12] Odds F. C. (2003). Synergy, antagonism, and what the chequerboard puts between them. J. Antimicrob. Chemoth 52, 1. doi: 10.1093/jac/dkg301 12805255

[B13] Rodrigues M. L. Albuquerque P. C. (2018). Searching for a change: The need for increased support for public health and research on fungal diseases. PloS Negl. Trop. Dis. 6, e0006479. doi: 10.1371/journal.pntd.0006479 PMC6001980 29902170

[B14] Rossato L. dos Santos M. C. Vitale R. G. de Hoog S. Ishida K. (2021). Alternative treatment of fungal infections: Synergy with non-antifungal agents. Mycoses 64, 232–244. doi: 10.1111/myc.13203 33098146

[B15] Sands M. Kron M. A. Brown R. B. (1985). Pentamidine: A review. Rev. Infect. Dis. 7, 625–6344. doi: 10.1093/clinids/7.5.625 3903942

[B16] Stokes J. M. MacNair C. R. Ilyas B. French S. Côté J.-P. Bouwman C.. (2017). Pentamidine sensitizes gram-negative pathogens to antibiotics and overcomes acquired colistin resistance. Nat. Microbiol. 2, 17028. doi: 10.1038/nmicrobiol.2017.28 28263303 PMC5360458

[B17] Sun T. Zhang Y. (2008). Pentamidine binds to tRNA through non-specific hydrophobic interactions and inhibits aminoacylation and translation. Nucleic Acids Res. 36, 1654–1664. doi: 10.1093/nar/gkm1180 18263620 PMC2275129

[B18] Thangamani S. Maland M. Mohammad H. Pascuzzi P. E. Avramova L. Koehler C. M.. (2017). Repurposing approach identifies auranofin with broad spectrum antifungal activity that targets Mia40-Erv1 pathway. Front. Cell. Infect. Microbiol. 7, 4. doi: 10.3389/fcimb.2017.00004 28149831 PMC5241286

[B19] Vandeputte P. Ferrari S. Coste A. T. (2012). Antifungal resistance and new strategies to control fungal infections. Int. J. Microbiol. 2012, 713687. doi: 10.1155/2012/713687 22187560 PMC3236459

[B20] Venturini T. P. Rossato L. Chassot F. Keller J. T. Piasentin F. B. Santurio J. M.. (2016). *In vitro* synergistic combinations of pentamidine, polymyxin b, tigecycline and tobramycin with antifungal agents against fusarium spp. J. Med. Microbiol. 65, 770–774. doi: 10.1099/jmm.0.000301 27357655

[B21] Wayne P. Clinical and Laboratory Standards Institute (2008). Reference method for broth dilution antifungal susceptibility testing of yeasts; approved standard-third edition M27-A3, Vol. 28. 14. (Wayne: Clinical and Laboratory Standards Institute)

[B22] Werner T. P. Amrhein N. Freimoser F. M. (2007). Specific localization of inorganic polyphosphate (poly p) in fungal cell walls by selective extraction and immunohistochemistry. Fungal Genet. Biol. 44, 845–852. doi: 10.1016/j.fgb.2007.01.008 17320430

[B23] Wiederhold N. P. Patterson T. F. Srinivasan A. Chaturvedi A. K. Fothergill A. W. Wormley F. L.. (2017). Repurposing auranofin as an antifungal: *In vitro* activity against a variety of medically important fungi. Virulence 8, 138–142. doi: 10.1080/21505594.2016.1196301 27268469 PMC5354159

[B24] Zhang Y. Bell A. Perlman P. S. Leibowitz M. J. (2000). Pentamidine inhibits mitochondrial intron splicing and translation in saccharomyces cerevisiae. RNA 6, 937–951. doi: 10.1017/S1355838200991726 10917591 PMC1369971

